# Risk of unemployment and work disability among refugee and non-refugee migrants with incident psychotic disorders in Sweden and Denmark

**DOI:** 10.1093/eurpub/ckad207

**Published:** 2023-12-19

**Authors:** Suborna Mastafa, Christopher J de Montgomery, Emma Pettersson, Marie Norredam, Allan Krasnik, Heidi Taipale, Ellenor Mittendorfer-Rutz, Alexis E Cullen

**Affiliations:** Division of Insurance Medicine, Department of Clinical Neuroscience, Karolinska Institutet, Stockholm, Sweden; Department of Public Health, Danish Research Centre for Migration, Ethnicity and Health (MESU), University of Copenhagen, Copenhagen, Denmark; Division of Insurance Medicine, Department of Clinical Neuroscience, Karolinska Institutet, Stockholm, Sweden; Department of Public Health, Danish Research Centre for Migration, Ethnicity and Health (MESU), University of Copenhagen, Copenhagen, Denmark; Section of Immigrant Medicine, Department of Infectious Diseases, University Hospital Hvidovre, Copenhagen, Denmark; Department of Public Health, Danish Research Centre for Migration, Ethnicity and Health (MESU), University of Copenhagen, Copenhagen, Denmark; Division of Insurance Medicine, Department of Clinical Neuroscience, Karolinska Institutet, Stockholm, Sweden; Department of Forensic Psychiatry, Niuvanniemi Hospital, Kuopio, Finland; School of Pharmacy, University of Eastern Finland, Kuopio, Finland; Division of Insurance Medicine, Department of Clinical Neuroscience, Karolinska Institutet, Stockholm, Sweden; Division of Insurance Medicine, Department of Clinical Neuroscience, Karolinska Institutet, Stockholm, Sweden

## Abstract

**Background:**

Unemployment and work disability are common among individuals with non-affective psychotic disorders (NAPDs) but it is unknown whether rates differ among migrants and native-born individuals. The present study aimed to compare the risk of these outcomes during the first 5 years of illness in non-refugee migrants, refugees and native-born individuals with NAPDs in Sweden and Denmark—two countries with different immigration policies and models of early psychosis care.

**Methods:**

Using national registers, we identified all individuals aged 18–35 years in Sweden and Denmark who received an incident NAPD diagnosis between 2006 and 2013 (*N* = 6750 and 8320, respectively). Cohorts were followed for 5 years to determine the days of unemployment and sickness absence (analyzed using zero-inflated negative binomial models) and the time to receipt of disability pension (analyzed using complementary log-log models).

**Results:**

Relative to their native-born peers, refugees and non-refugee migrants in Sweden and non-refugee migrants in Denmark were significantly less likely to have zero unemployment days (OR range: 0.54–0.72) and all migrant groups experienced more unemployment days (IRR range: 1.26–1.37). Results were largely unchanged after adjustment for sociodemographic and clinical factors. In the adjusted model, both Swedish migrant groups and refugees in Denmark were more likely to experience zero sickness absence days than native-born individuals (OR range: 1.48–1.56). Only refugees in Denmark were at greater risk of disability pension.

**Conclusions:**

Non-refugee migrants and refugees with NAPDs in both Sweden and Denmark are particularly vulnerable to experiencing unemployment. Targeted interventions may help to reduce these disparities and promote long-term work ability among migrant groups.

## Introduction

Psychotic disorders (of which schizophrenia is the most common and severe) are a heterogeneous group of mental disorders with a lifetime prevalence of 7.5 per 1000 individuals.^1^ Due to their early age of onset, and typically chronic course, these disorders can severely limit work ability, with nationwide studies in Europe and Asia reporting low employment rates (ranging 4–25%) in this population.^2–4^ Social protection systems, such as sickness absence and disability pension benefits, can provide income for individuals with decreased work capacity due to mental disorders. Sickness absence is commonly used for time-limited work incapacity, intended to facilitate recovery, with disability pension used when illness is likely to permanently limit occupational functioning. Whilst the use of these social protection policies in psychosis populations has been less commonly studied than unemployment, data from Sweden,^3^ Northern Finland^5^ and France,^6^ suggest that as many as 80% of individuals with schizophrenia receive disability pension, and that among those who are able to work, sickness absence is common.^7^

Given the high rates of labour market marginalization among individuals with psychosis, it is important to determine whether there are vulnerable subgroups who might benefit from targeted interventions to improve work ability. Mental disorders are highly prevalent among migrants,^8^^,^^9^ particularly refugees,^8^^,^^10^^,^^11^ and the risk of developing psychotic disorders is elevated in these groups compared with the general population.^12^ Experiences common to both migrant groups (e.g. discrimination, acculturation, language barriers, limited healthcare access and lack of social support) may explain these findings, whilst for refugees, traumatic events experienced pre- and post-migration likely play an important role.^10^ Whilst studies have shown that in the general population, migrant groups (particularly refugees) experience higher levels of labour market marginalization than native-born individuals,^13–15^ it is unclear whether this is true among those with psychosis. Two studies of individuals with first-episode psychosis in Canada^16^ and Switzerland^17^ observed no significant differences in employment rates among migrant and native-born individuals at 24 and 36 months of follow-up, respectively. However, an Italian study reported that migrants with psychosis were more likely than their native-born peers to suspend their work/educational activities during the first 12 months of illness (although they were also more likely to return to work during this period).^18^ Although these studies suggest that migrant status is not associated with reduced employment among individuals with psychosis, the impact on sickness absence and disability pension is unknown. Moreover, it is important to distinguish between non-refugee and refugee migrants, given that traumatic events (more commonly experienced by refugees)^19^ are associated with labour market marginalization in other populations.^20^^,^^21^

To address these knowledge gaps, the present study used the Swedish and Danish nationwide registers to compare risk of unemployment, sickness absence and disability pension among non-refugee migrants, refugees and native-born individuals with psychosis. This international comparison is important because these countries differ substantially in terms of the availability of psychosis early intervention (EI) services, which have become widespread in Denmark over the past two decades,^22^ but are limited in Sweden. Given that support with education and employment is a core component the EI model, we might expect work ability for individuals with psychosis to be improved in countries where EI is widely available. In addition, there were substantial differences in immigration and integration policies during the study period, with Sweden adopting a more ‘inclusionist’ approach (characterised by a higher immigrant population and an emphasis on creating equal opportunities) while a more ‘restrictive’ policy (including greater barriers to accessing financial support) was implemented in Denmark.^23^ These policies could potentially influence disparities between migrant groups and native-born individuals.

We aimed to determine whether there are differences in labour market marginalization in refugees and non-refugee migrants compared with native-born individuals in the 5 years following the first diagnosis of a psychotic disorder and if patterns were similar in Sweden and Denmark. Considering previous studies showing substantial differences in labour market participation between schizophrenia/schizophrenia-spectrum disorders and bipolar/major depressive disorder,^3^^,^^4^ we studied individuals experiencing incident non-affective psychotic disorders (NAPDs).

## Methods

### Data sources

Data were obtained from the Swedish and Danish national registers detailed in Supplementary table S1. Within each country, registers were linked by the unique personal (pseudonymised) identification numbers assigned to all residents at birth or immigration.

### Design and study population

The study populations comprised individuals aged 18–35 years in Sweden and Denmark who received their first NAPD diagnosis in inpatient/specialist outpatient services between the 1 January 2006 and 31 December 2013. Diagnoses were defined as the main diagnosis assigned at discharge/physician contact (for inpatient/outpatient treatments, respectively) according to International Classification of Diseases—version 10 (ICD-10: F20-F29).^24^ To capture incident NAPD cases, we required that individuals were registered as resident in Sweden/Denmark on the 31 December in the year of cohort entry and each of the three previous calendar years, and that they had no inpatient/outpatient contacts for NAPD in the previous 3 years (1080 days). Individuals with recorded purchases of antipsychotic medication (Anatomic Therapeutic Chemical classification [ATC] codes N05A, excluding N05AN) prior to cohort entry were excluded. Due to the fact that the Swedish Prescribed Drug Register was only established in July 2005, we used an exclusion period of 15–3 months prior to cohort entry for antipsychotic purchases (providing a minimum observation period of 6 months for all cohort members), purchases in the 3 months preceding diagnosis were considered part of the first treatment episode. Because disability pension is often permanent and precludes receipt of unemployment and sickness absence benefits, we excluded those who received disability pension in the year before cohort entry.

### Exposure measures

Refugees were defined as individuals whose grounds for residence as registered with the Swedish/Danish immigration authorities was ‘refugee status’ or ‘family reunification with a refugee’. As grounds of residence data were only available from 1993 in Denmark, immigrants arriving from the major refugee-sending countries in the period 1986–92 were defined as refugees. All other migrants, irrespective of country-of-birth, were classified as non-refugee migrants. Individuals born in Sweden/Denmark were classified as native-born.

### Outcome measures

The study populations were followed for five calendar years, with three occupational outcome measures examined: (i) the number of registered unemployment days, (ii) gross number of sickness absence days and (iii) time until disability pension was granted (measured in discrete calendar years due to the availability of data for analysis in Danish registers). Due to differences in the benefits structure across countries (detailed in Supplementary table S2), in Sweden, we considered only long-term sickness absence (i.e. spells > 30 days) to provide harmonization with the Danish payment system.

### Covariates

Model covariates (defined in Supplementary table S1) included age, gender, family situation, educational level, region of residence, NAPD diagnosis at first contact, prior treatment for other mental disorders, prior treatment for somatic conditions, calendar year at cohort entry (included to account for any changes in benefit payment regulations over time) and labour market income at baseline (included in sensitivity analyses only).

### Statistical methods

Statistical analyses were performed in Sweden and Denmark separately using R version 4.2.1 and 4.0.4, respectively. Sickness absence and unemployment days were analyzed with zero-inflated negative binomial regression models, appropriate for analyzing over-dispersed count data with excess zero counts, using the ‘pscl’ package. The excess zeros are assumed to be generated by a separate structural process from the count values and the two processes are modelled separately with a logistic and a negative-binomial regression. A logistic regression estimates the odds of excess/structural zero values and a negative binomial regression is used to model the remaining count process.^25^^,^^26^ Adjusted odds ratios (OR) and incident rate ratios (IRR) are presented along with 95% confidence intervals (CI). Unemployment and sickness absence days were modelled in relation (offset) to the total number of observed days from the 1st of January in the year of cohort entry until death/emigration or the end of the 5-year follow-up period. Plots showing the combined results from the two components of the zero-inflated negative binomial models were generated using ‘emmean’ and ‘ggplot2’ packages. Sensitivity analyses were conducted for both outcomes where individuals were followed until death/emigration, the end of the 5-year follow-up, or until the end of the calendar year when disability pension was first granted. Here, unemployment and sickness absence outcomes were recomputed excluding outcomes occurring after the year when disability pension was first granted. Consequently, individuals who were granted disability pension in the cohort entry year were not included in sensitivity analyses. Separate sensitivity analyses were also performed for sickness absence days with baseline labour market income included as a covariate.

Risk of disability pension during the 5-year follow-up was modelled using a complementary log-log (cloglog) model for interval-censored survival using the ‘survival’ package yielding hazard ratios (HR) with 95% CIs. This model is a discrete analogue to the continuous Cox proportional hazards model and is appropriate when exact timings of event occurrences are unknown and when events are instead known to have occurred during particular time intervals.^27^ Kaplan–Meier curves generated using ‘ggplot2’.

## Results

The Swedish cohort consisted of 6750 individuals, including 5066 (75.1%) Swedish-born, 772 (11.4%) non-refugee migrants and 912 (13.5%) refugees. The Danish cohort comprised 8320 individuals, of whom 6951 (83.5%) were native-born, 860 (10.3%) were non-refugee migrants and 509 (6.1%) were refugees. Sample characteristics are provided by country and population group in table 1; region-of-birth and duration-of-residence data are provided for migrant groups in Supplementary table S3. In both Sweden and Denmark, individuals aged 18–23 years comprised the largest age group (39.6% and 49.9%, respectively). Males formed the majority in Sweden (63.8%) and Denmark (60.4%), with the highest proportion observed among refugees in both countries. In the total populations, few individuals were married/cohabiting (7.5% in Sweden and 5.0% in Denmark), although in both countries, this was more common among non-refugee migrants. Across all groups, between 51% and 65% received any labour market income in the year prior to first diagnosis.

**Table 1:** Sociodemographic and clinical characteristics of individuals with NAPDs in Sweden and Denmark

	Sweden								Denmark							
	Total (*N* = 6750)		Swedish-born (*N* = 5066)		Non-refugee migrants (*N* = 772)		Refugees (*N* = 912)		Total (*N* = 8320)		Danish-born (*N* = 6951)		Non-refugee migrants (*N* = 860)		Refugees (*N* = 509)	
	
	*n*	(%)	*n*	(%)	*n*	(%)	*n*	(%)	*n*	(%)	*n*	(%)	*n*	(%)	*n*	(%)
Age (years)^a^																
18–23	2673	(39.6)	2120	(41.8)	168	(21.8)	385	(42.2)	4150	(49.9)	3689	(53.1)	279	(32.4)	182	(35.8)
24–29	2222	(32.9)	1651	(32.6)	250	(32.4)	321	(35.2)	2458	(29.5)	1981	(28.5)	291	(33.8)	186	(36.5)
30–35	1855	(27.5)	1295	(25.6)	354	(45.9)	206	(22.6)	1712	(20.6)	1281	(18.4)	290	(33.7)	141	(27.7)
Gender^b^																
Male	4305	(63.8)	3249	(64.1)	416	(53.9)	640	(70.2)	5027	(60.4)	4167	(59.9)	498	(57.9)	362	(71.1)
Female	2445	(36.2)	1817	(35.9)	356	(46.1)	272	(29.8)	3293	(39.6)	2784	(40.1)	362	(42.1)	147	(28.9)
Family situation^b^																
Single	6243	(92.5)	4868	(96.1)	597	(77.3)	778	(85.3)	7902	(95.0)	6686	(96.2)	752	(87.4)	464	(91.2)
Married/cohabiting	507	(7.5)	198	(3.9)	175	(22.7)	134	(14.7)	418	(5.0)	265	(3.8)	108	(12.6)	45	(8.8)
Type of residence region^b^																
Cities	3256	(48.2)	2275	(44.9)	443	(57.4)	538	(59.0)	3719	(44.7)	2947	(42.4)	505	(58.7)	267	(52.5)
Towns/Suburbs	2481	(36.8)	1942	(38.3)	254	(32.9)	285	(31.3)	2486	(29.9)	2152	(31.0)	192	(22.3)	142	(27.9)
Rural areas	1013	(15.0)	849	(16.8)	75	(9.7)	89	(9.8)	2115	(25.4)	1852	(26.6)	163	(19.0)	100	(19.6)
Educational level^b^																
Compulsory	3205	(47.5)	2223	(43.9)	396	(51.3)	586	(64.3)	5912	(71.1)	4966	(71.4)	552	(64.3)	394	(77.4)
High school	2100	(31.1)	1717	(33.9)	173	(22.4)	210	(23.0)	2090	(25.1)	1735	(25.0)	253	(29.5)	102	(20.0)
University	1445	(21.4)	1126	(22.2)	203	(26.3)	116	(12.7)	317	(3.8)	250	(3.6)	54	(6.3)	13	(2.6)
Labour market income^b^																
None	2558	(37.9)	1777	(35.1)	334	(43.3)	447	(49.0)	3110	(37.4)	2570	(37.0)	307	(35.7)	233	(45.8)
Any	4192	(62.1)	3289	(64.9)	438	(56.7)	465	(51.0)	5210	(62.6)	4381	(63.0)	553	(64.3)	276	(54.2)
NAPD diagnosis first contact																
Schizophrenia	459	(6.8)	327	(6.5)	50	(6.5)	82	(9.0)	2967	(35.7)	2519	(36.2)	270	(31.4)	178	(35.0)
Schizotypal	94	(1.4)	88	(1.7)	<10	NR	<10	NR	1632	(19.6)	1482	(21.3)	121	(14.1)	29	(5.7)
Delusional disorder	565	(8.4)	416	(8.2)	>70	NR	>70	NR	755	(9.1)	582	(8.4)	95	(11.0)	78	(15.3)
Brief psychotic episode	2423	(35.9)	1845	(36.4)	275	(35.6)	303	(33.2)	2089	(25.1)	1638	(23.6)	288	(33.5)	163	(32.0)
Other	3209	(47.5)	2390	(47.2)	369	(47.8)	450	(49.3)	877	(10.5)	730	(10.5)	86	(10.0)	61	(12.0)
Treatment for mental disorder^c^																
None	3485	(51.6)	2510	(49.5)	462	(59.8)	513	(56.3)	5224	(62.8)	4307	(62.0)	569	(66.2)	348	(68.4)
Any	3265	(48.4)	2556	(50.5)	310	(40.2)	399	(43.8)	3096	(37.2)	2644	(38.0)	291	(33.8)	161	(31.6)
Treatment for somatic conditions^c^																
None	2866	(42.5)	2168	(42.8)	337	(43.7)	361	(39.6)	3096	(37.2)	2608	(37.5)	313	(36.4)	175	(34.4)
Any	3884	(57.5)	2898	(57.2)	435	(56.3)	551	(60.4)	5224	(62.8)	4343	(62.5)	547	(63.6)	334	(65.6)
Cohort entry year																
2006	806	(11.9)	618	(12.2)	96	(12.4)	92	(10.1)	859	(10.3)	718	(10.3)	83	(9.7)	58	(11.4)
2007	759	(11.2)	560	(11.1)	94	(12.2)	105	(11.5)	902	(10.8)	720	(10.4)	106	(12.3)	76	(14.9)
2008	823	(12.2)	622	(12.3)	88	(11.4)	113	(12.4)	950	(11.4)	787	(11.3)	107	(12.4)	56	(11.0)
2009	821	(12.2)	622	(12.3)	85	(11.0)	114	(12.5)	1007	(12.1)	824	(11.9)	115	(13.4)	68	(13.4)
2010	837	(12.4)	620	(12.2)	96	(12.4)	121	(13.3)	1063	(12.8)	886	(12.7)	108	(12.6)	69	(13.6)
2011	849	(12.6)	650	(12.8)	99	(12.8)	100	(11.0)	1138	(13.7)	968	(13.9)	102	(11.9)	68	(13.4)
2012	913	(13.5)	662	(13.1)	106	(13.7)	145	(15.9)	1174	(14.1)	995	(14.3)	121	(14.1)	58	(11.4)
2013	942	(14.0)	712	(14.1)	108	(14.0)	122	(13.4)	1227	(14.7)	1053	(15.1)	118	(13.7)	56	(11.0)

Notes: Missing data Danish cohort: education level (*n *=* *1). NR, not reported to prevent determination of cells with counts < 10.

a Measured during year of cohort entry.

b Measured on 31 December in the calendar year prior to cohort entry.

c Measured in the three relative years (1080 days) prior to cohort entry date.

Some notable differences across countries were observed. In the total Swedish cohort, 21.4% had completed university education compared with only 3.8% in Denmark, potentially reflecting the younger age of the latter cohort. Moreover, whilst schizophrenia was the most common diagnosis in the total Danish population (35.7%), other psychotic disorders (ICD-10: F24–F29) were most common in Sweden (47.5%). Treatment for other mental disorders in the 3 years prior to cohort entry was also somewhat more common in Sweden than in Denmark (48.4% vs. 37.2%, respectively) and in both countries was more common among native-born individuals.

### Unemployment and sickness absence

Descriptive statistics for unemployment and long-term sickness absence (Supplementary table S4) showed that individuals in the Swedish cohort more commonly experienced at least one unemployment day compared with Denmark (65.0% vs. 31.1%, respectively) with the same pattern observed for long-term sickness absence (38.8% vs. 25.4%, respectively). Moreover, the median number of unemployment and sickness absence days across all groups was far higher across all Swedish groups when compared with their Danish counterparts. Across both countries, native-born individuals were least likely to be unemployed whilst refugees were least likely experience long-term sickness absence.

Estimates from the crude zero-inflated negative binomial models for unemployment and sickness absence days are shown in table 2, in the binary logistic component of the zero-inflated negative binomial model, the coefficients should be interpreted as the odds of observing ‘excess’ or ‘structural’ zero responses, which are beyond that which can be explained by a typical negative binomial distribution. Compared with native-born individuals, the odds of experiencing zero unemployment days were significantly lower among both non-refugee migrants (OR = 0.72, 95% CI = 0.61–0.85) and refugees (OR = 0.54, 95% CI = 0.46–0.63) in Sweden, and among non-refugee migrants in Denmark (OR = 0.67, 95% CI = 0.57–0.77). In the count component of the model (which simultaneously estimates the rates of unemployment days among those who may experience unemployment), the average number of days was significantly higher among non-refugee migrants and refugees in both Sweden (IRR = 1.28 and 1.37, respectively) and Denmark (IRR = 1.31 and 1.26) relative to their native-born peers. In contrast, whilst the odds of experiencing zero sickness absence days (excluding spells ≤ 30 days) were lower among non-refugee migrants in Denmark compared with their native-born peers (OR = 0.84, 95% CI = 0.72–0.98) the odds were higher among refugees in Sweden (OR = 1.76, 95% CI = 1.51–2.05). In both countries, no differences were observed in the count components of the models. Adjustment for sociodemographic and clinical covariates resulted in some changes to the pattern of results for sickness absence (table 2).

**Table 2:** Results of zero-inflated negative binomial models applied to days of unemployment and long-term sickness absence during the 5-year follow-up

	Sweden								Denmark							
	**Zero-inflated component** ^a^				**Count component** ^b^				**Zero-inflated component** ^a^				**Count component** ^b^			
	Crude		Adjusted		Crude		Adjusted		Crude		Adjusted		Crude		Adjusted	
	OR	(95% CI)	OR	(95% CI)	IRR	(95%CI)	IRR	(95%CI)	OR	(95% CI)	OR	(95% CI)	IRR	(95%CI)	IRR	(95%CI)
Unemployment days																
Native-born																
Non-refugee migrant	0.72	(0.61–0.85)	0.63	(0.53–0.75)	1.28	(1.17–1.41)	1.15	(1.04–1.27)	0.67	(0.57–0.77)	0.78	(0.66–0.91)	1.31	(1.14–1.50)	1.20	(1.05–1.38)
Refugee	0.54	(0.46–0.63)	0.51	(0.43–0.60)	1.37	(1.26–1.49)	1.32	(1.21–1.44)	0.97	(0.79–1.18)	1.16	(0.93–1.43)	1.26	(1.03–1.53)	1.17	(0.96–1.41)
Sickness absence days																
Native-born																
Non-refugee migrant	1.16	(1.00–1.36)	1.50	(1.26–1.79)	1.03	(0.92–1.16)	0.99	(0.88–1.11)	0.84	(0.72–0.98)	1.17	(0.98–1.39)	1.01	(0.92–1.11)	0.99	(0.90–1.09)
Refugee	1.76	(1.51–2.05)	1.56	(1.32–1.85)	1.08	(0.96–1.21)	1.07	(0.95–1.21)	1.23	(0.99–1.53)	1.48	(1.17–1.88)	0.96	(0.84–1.10)	0.95	(0.83–1.09)

Notes: OR, odds ratio; CI, confidence interval; IRR, incidence rate ratio. Adjusted models included age, gender, family situation, region of residence, education level, psychotic disorder diagnosis at first contact, previous treatment for any mental disorder, previous treatment for somatic conditions and calendar year at cohort entry.

a Zero-inflated component modelled using binary logistic regression to estimate the odds of excess/structural zero values.

b Count component modelled with negative binomial regression to estimate the rate of remaining (non-structural zero) days.

**Figure 1:**
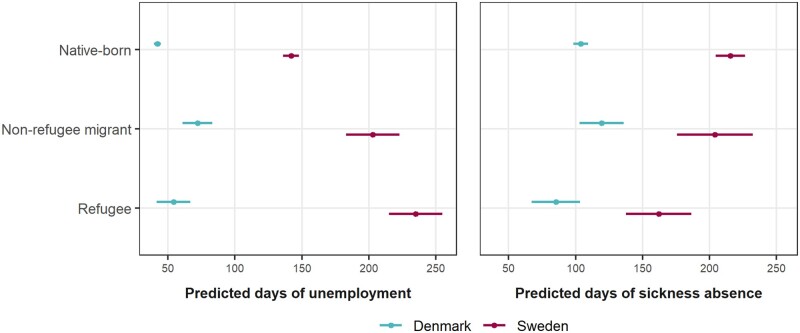
Estimated marginal means predicting unemployment and sickness absence days during the 5-year follow-up derived from crude zero-inflated negative binomial models. Overall estimates along with 95% confidence intervals, by population group and country, accounting for both the zero-inflated and count components of the models, are presented

Figure 1 shows the combined effect of two crude model components for these outcomes by country. Overall, compared with native-born individuals, predicted days of unemployment were higher among all migrant groups except refugees in Denmark, whilst predicted days of sickness absence were lower only among refugees (and to a greater extent in Sweden).

In sensitivity analyses (Supplementary table S5) the effects of population group on long-term sickness absence days were somewhat attenuated when labour market income at baseline was included as an additional covariate. Here, the higher odds of zero sickness absence days in the Danish refugee group was no longer significant (results for Swedish non-refugee migrants and refugees retained significance). In separate models censored for receipt of disability pension, there were no changes to the overall pattern of results for unemployment and sickness absence outcomes.

### Disability pension

Receipt of disability pension is shown in Supplementary table S4 by country and population group. Overall, disability pension receipt was less common in Denmark (20.0%) than Sweden (31.3%), and in both countries, receipt was most common among refugees. In the crude model (table 3 and Supplementary figure S1), refugees in Denmark were at higher risk of disability pension compared with their native-born peers during the follow-up (HR = 1.63, 95% 1.37–1.92). This effect was slightly attenuated after adjustment for sociodemographic and clinical covariates. In contrast, non-refugee migrants in Denmark and Swedish refugee and non-refugee migrants did not differ to native-born individuals.

**Table 3:** Complementary log-log model applied to time to disability pension during the 5-year follow-up

	Sweden				Denmark			
	Crude		Adjusted		Crude		Adjusted	
	HR	(95% CI)	HR	(95% CI)	HR	(95% CI)	HR	(95% CI)
Native-born								
Non-refugee migrant	0.88	(0.76–1.02)	1.08	(0.93–1.25)	1.06	(0.90–1.23)	0.97	(0.82–1.14)
Refugee	1.09	(0.96–1.23)	1.01	(0.89–1.15)	1.63	(1.37–1.92)	1.26	(1.06–1.50)

Note: HR, hazard ratio; CI, confidence interval.

## Discussion

To our knowledge, this is the first study to compare multiple measures of labour market marginalization among non-refugee migrants, refugees and native-born individuals with NAPDs. We observed that both migrant groups experienced higher levels of unemployment during the first 5 years of illness relative to their Swedish-born and Danish-born peers. Findings for long-term sickness absence were more mixed, and only refugees in Denmark were at increased risk of receiving disability pension. In general, results were largely unchanged after accounting for a range of sociodemographic and clinical factors.

Before discussing the impact of migrant status on outcomes, we consider the country-level differences that we observed. Whilst rates of unemployment, long-term sickness absence and disability pension among individuals with NAPDs were high in both countries, all outcomes were markedly higher in Sweden. For disability pension, this is likely because temporary payments were not included in the Danish measure and (since 2013) the age at which one can receive permanent disability pension is higher than that in Sweden (detailed in Supplementary Information). However, country-level differences were most apparent for unemployment (experienced by 65.0% of individuals with NAPD in Sweden and 31.1% of those in Denmark), which is unexpected given that unemployment measures were consistent across countries. One possible explanation is that disease burden among individuals with NAPD is lower in Denmark, potentially due to the high availability of psychosis EI services which provide support with education and employment.^28^ Indeed, clinical trials indicate that EI treatment is associated with increased participation in education/employment relative to standard care.^29^ It is important to note, however, that as we did not standardize outcomes to account for differences in population characteristics, rates are not directly comparable across countries. Moreover, national differences and changes in immigration and integration policies during the study period^23^ could influence the extent to which migrants are eligible to receive unemployment and work disability payments.

Despite the country-level differences in unemployment rates, the effect of migrant/refugee status on this outcome was largely consistent across Sweden and Denmark. Non-refugee migrants in both countries and refugees in Sweden were significantly less likely to have zero unemployment days and were estimated to experience a higher number of unemployment days over the 5-year follow-up. These findings are in contrast to studies from Canada^16^ and Switzerland,^17^ which found no significant differences in employment rates among migrant and native-born individuals with first-episode psychosis at 24 and 36 months of follow-up, respectively. This difference in findings could be attributable to our longer follow-up period; however, as we examined registered unemployment (rather than self-reported employment), the findings are not directly comparable. Our findings are, however, consistent with the elevated rates of unemployment that have been observed in Nordic countries among non-refugee migrants and refugees within the general population,^14^^,^^30^ and among those with common mental disorders.^31^ Somatic comorbidities, language proficiency, educational levels, work-related skills and discrimination likely contribute to unemployment in both migrant groups. Whilst we attempted to account for some of these factors, residual confounding is possible. Irrespective of the reasons, it is vital that psychosis services are aware of the important disparities faced by these groups and provide targeted support to enable migrant groups to achieve better long-term functioning.

Migrant groups in Sweden were observed to have higher likelihood of experiencing zero sickness absence days (excluding spells ≤ 30 days) during the follow-up relative to their native-born peers. In Denmark, opposing patterns were found for non-refugee migrants (who had significantly lower odds of zero sickness absence days in the crude model) and refugees (who had significantly higher odds but only in the adjusted model). Whilst we are not aware of any previous studies comparing this outcome among migrants and native-born individuals with NAPDs, our results are partially in line with a previous study in which non-refugee migrants and refugees with common mental disorders were found to be at lower risk of sickness absence spells >180 days relative to Swedish-born individuals with these disorders.^31^ One potential explanation for the lower likelihood of long-term sickness absence among migrant groups in Sweden and refugees in Denmark is that these groups are at greater risk of long-term exclusion from the labour market,^14^ and previous employment is a prerequisite for receiving this benefit. Indeed, we observed that in the year prior to NAPD onset, having any labour market income was less common among both migrant groups in Sweden and refugees in Denmark relative to native-born individuals. However, when we accounted for labour market income at baseline, the findings for Swedish refugees and non-refugee migrants were only slightly attenuated.

We found that only refugees in Denmark experienced higher risk of disability pension compared with their native-born peers. Previous studies from Sweden have observed lower risk of this outcome among both refugee and non-refugee migrants treated for common mental disorders,^31^ but higher risk among non-refugee migrants within the general population.^14^ In both studies, estimates in migrant groups varied according to country of birth and duration of residence; differences in the composition of these migrant groups in the present study may therefore explain why our findings differ to some previous studies and why findings were inconsistent across countries.

### Strengths and limitations

Our use of large-scale, high-quality, national registers from Sweden and Denmark enabled us to capture a wide range of potential confounders and eliminate loss to follow-up (except in cases of death or emigration). Moreover, we examined multiple measures of labour market marginalization, which is important given the potential for substitution effects (i.e. individuals ineligible for one benefit may receive another). However, some limitations should be noted. First, levels of unemployment and work disability may be underestimated in register data. Second, whilst we adjusted for a range of clinical factors (including diagnosis at first contact and prior healthcare contacts for other mental and somatic conditions), we lack information on illness severity/course. As such, residual confounding is likely. Third, the sickness absence benefits that we measured pertain to those who are (or have been) employed, and therefore we did not capture sickness spells that impact the ability to complete education, which may be particularly relevant to this age group. Finally, as our native-born groups included second-generation migrants, we may have underestimated the levels of labour market marginalization among migrant groups.

## Conclusions

Using national data from two Nordic countries, we have shown that individuals with NAPDs experience high levels of labour market marginalization. Moreover, within this population, non-refugee migrants and refugees are particularly vulnerable to experiencing unemployment, even after accounting for clinical and sociodemographic factors. Logistical and cultural barriers to attending and engaging with mental health services, the absence of translators and experiences of discrimination may impact the type and quality of care received by migrants with NAPDs, which could in turn have downstream consequences for their functional recovery and work ability. Whilst we are unable to disentangle the complex factors that may further exacerbate the risk of unemployment among migrant groups, our findings have important implications for mental health services who should be aware that additional support is needed for migrants to achieve better long-term outcomes. Indeed, an important avenue for future research is to investigate whether migrant groups can equally benefit from employment-focused interventions (e.g. individual placement and support) commonly implemented within psychosis EI services. With regard to work disability, whilst sickness absence and disability pension are markers of impaired functioning, it is important to note that receipt of these benefits indicates that individuals are receiving appropriate financial support when work ability is reduced due to illness. As such, the lower rates of sickness absence benefits and lack of differences in disability pension that we observed in migrants and refugees (populations which we might expect to have a high level of sickness burden) relative to their native-born peers could indicate barriers to accessing these support systems.

## Supplementary Material

ckad207_Supplementary_Data

## Data Availability

The data used in this study cannot be made publicly available due to privacy regulations. According to the General Data Protection Regulation, the Swedish law SFS 2018:218, the Swedish Data Protection Act, the Swedish Ethical Review Act and the Public Access to Information and Secrecy Act, these types of sensitive data can only be made available for specific purposes, including research, which meet the criteria for access to this sort of sensitive and confidential data as determined by a legal review. Readers may contact Professor Kristina Alexanderson (kristina.alexanderson@ki.se) regarding the data.
