# Supplementary material for: Risk of unemployment and work disability among refugee and non-refugee migrants with incident psychotic disorders in Sweden and Denmark
Source: Eur J Public Health. 2023 Dec 19;34(1):129–35. doi: 10.1093/eurpub/ckad207 (PMC10843956; doi:10.1093/eurpub/ckad207)
Supplement: ckad207_Supplementary_Data [file ckad207_supplementary_data.pdf]

Supplementary Material

Mastafa S, de Montgomery CJ, Pettersson E, Norredam M, Krasnik A, Taipale H, Mittendorfer-Rutz E, Cullen AE. Risk of unemployment and work disability among refugee and non-refugee migrants with incident psychotic disorders in Sweden and Denmark.

Tables

Table S1 Data sources and associated variables in Sweden and Denmark 2
Table S2 Current benefit payment structure in Sweden and Denmark and availability of data in associated registers 4
Table S3 Region of birth and duration of residence for non-refugee migrant and refugee populations 5
Table S4 Descriptive statistics for unemployment and work disability outcomes during the five-year follow-up among individuals with non-affective psychotic disorders in Sweden and Denmark........ 6
Table S5 Sensitivity analyses for sickness absence with labour market income at baseline included as an additional covariate 7
Table S6 Sensitivity analyses for unemployment and sickness absence outcomes accounting for receipt of disability pension during the five-year follow-up........ 8

Figures

Figure S1. Kaplan-Meier estimated survival curves. Proportion not receiving disability pension during the five-year follow-up with 95% confidence intervals, by population group and country. 9

Table S1 Data sources and associated variables in Sweden and Denmark

Variable	Definition	Swedish register	Danish Register
**Inclusion criteria**			
Diagnosis criteria	Main diagnosis of NAPD (ICD-10 codes F20-29), received in inpatient or specialist outpatient services at discharge or first assessment with physician, respectively, between the 1st of January 2006 and 31st of December 2013 inclusive	NPR	NPR
Prior diagnosis criteria	No recorded inpatient or outpatient contacts with a main diagnosis of NAPD in the three years (1080 days) prior to cohort entry	NPR	NPR
Residency criteria	Registered as resident in Sweden/Denmark on the 31st of December in the year of cohort entry and each of the three previous calendar years	LISA	BEF
Medication criteria	Purchases of antipsychotic medication (Anatomic Therapeutic Chemical classification [ATC] codes N05A, excluding N05AN) in 15 months to 3 months prior to cohort entry	PDR	DPR
**Exposures**			
Refugee status	Grounds for residence in Sweden/Denmark registered with immigration authorities as “refugee status” or “family reunification with a refugee”, categorised as no vs. yes	STATIV	IEPE, OPHG
Non-refugee status	Born in country other than host country, categorised as no vs. yes	LISA	BEF
**Outcomes**			
Unemployment	Number of registered unemployment days during the five calendar years following the year of first NAPD diagnosis	LISA	IDAP
Sickness absence	Gross sickness absence days during the five calendar years following the year of first NAPD diagnosis	MiDAS	SGDP
Disability pension	Time, measured in discrete calendar year intervals, until first granting of disability pension during the five calendar years following the first NAPD diagnosis	MiDAS	IND, SOCP
**Covariates**			
Age	Years of age during the calendar year of cohort entry, categorised as 18-23 years vs. 24-29 years vs. 30-35 years	LISA	BEF
Gender	Measured on the 31st of December in the year prior to cohort entry, categorised as men vs. women	LISA	BEF
Family situation	Measured on the 31st of December in the year prior to cohort entry, categorised as married/cohabiting vs. other	LISA	BEF
Type of residence region	Measured on the 31st of December in the year prior to cohort entry, categorised as cities vs. towns/suburbs vs. rural, according to EUROSTAT’s degree of urbanization [DEGURBA] classification of local administrative units	LISA	BEF
Level of education	Measured on the 30th September (Denmark) / 31st of December (Sweden) in the year prior to cohort entry, categorised as compulsory vs. high school vs. college/university	LISA	BUE
Labour market income	Any income from work received in the calendar year prior to cohort entry, categorised as no vs. yes	LISA	IND
Treatment for psychiatric disorder	Any inpatient/specialist outpatient treatment with a main diagnosis of any non-psychotic psychiatric disorder (ICD-10 codes F00-F99, excluding F20-29) during the three relative years (1080 days) prior to cohort entry, categorised as no vs. yes	NPR	NPR
Treatment for somatic conditions	Any inpatient/specialist outpatient treatment with a main diagnosis of any somatic condition (ICD-10 codes A-Z, excluding F, O80, X60-X84, Y10-Y34, and Z) during the three relative years (1080 days) prior to cohort entry, categorised as no vs. yes	NPR	NPR

NAPD diagnosis category	NAPD diagnosis at first contact (assigned at discharge/contact for inpatient/outpatient treatments, respectively), categorised as schizophrenia (ICD-10: F20) vs. schizotypal disorder (ICD-10: F21) vs. delusional disorder (ICD-10: F22) vs. acute or transient psychotic disorder (ICD-10: F23) vs. other (ICD-10: F24-F29)	NPR	NPR
**Other variables**			
Death	Death during five-year study period, categorised as no vs. yes	CDR	DODSAASG
Emigration	Emigration during five-year study period, categorised as no vs. yes	LISA	BEF
Region of birth [a]	Defined according to the United Nations Standard Country or Area Codes for Statistical Use [1] as Africa vs. Western Asia vs. Other Asia vs. Europe	LISA	IEPE
Duration of residence [a]	Duration of residence in host country, categorised as 0-5 years vs. 6-10 years vs. 11+ years	LISA	VNDS

[a] Determined for refugees and non-refugee migrant groups.

Abbreviations: ACT: Anatomic Therapeutic Chemical; BEF: Population Registry; BUE: Population Education Register; CDR: Cause of Death Register; DODSAASG: Register of Causes of Death; DPR: Drug Prescription Registry; ICD-10: International Classification of Diseases – version 10; IDAP: Database for Labour Market Research; IEPE: Immigrants and descendants; IND: Income Registry; LISA: Longitudinal Integration Database for Health Insurance and Labor Market Studies; MiDAS: Micro-Data for Analyses of Social Insurance; NAPD: non-affective psychotic disorder; NPR: National Patient Register; OPHG: Grounds of Residence (including supplementary dataset from the Immigration Agency covering 1993-1996); PDR: Prescribed Drug Register; SGDP: Sickness Absence Register; SOCP: Social Pensions; STATIV: Longitudinal Database for Integration Studies; VNDS: Historical migrations.

Table S2 Current benefit payment structure in Sweden and Denmark and availability of data in associated registers

	Sweden	Denmark
**Unemployment benefits**	Individuals are entitled to income-related levels of unemployment benefit (from age 16 years) or basic levels (if aged ≥20 years and having no recent job income), when registered as a job seeker at the Swedish Public Employment Service. Basic unemployment applies to everyone in Sweden who has worked there prior to unemployment and fulfils a working condition. Income-related benefit is available to those who have additionally been a member of an unemployment insurance fund for at least 12 consecutive months. Our outcome measure, days of registered unemployment, captures individuals who received cash benefits, as well as individuals who registered as unemployed but who were ineligible for benefits.	Unemployment benefit is available to individuals who are aged ≥18 years and have been a member of a recognised unemployment insurance fund for at least one year (those <18 years can enter an insurance fund if they have completed at least 18 months of vocational training). Individuals who are not a member of an unemployment insurance fund can receive cash benefits ('kontanthjælp') at either the full (if aged ≥30 years) or youth rate (if aged <30 years and have a qualifying educational level). For both benefit types, individuals must be registered as unemployed with the municipal employment agency. The national registers capture the proportion of the year spent registered as unemployed and therefore includes individuals who receive both payment types.
**Sickness absence** [1]	All individuals aged ≥16 years, with an income above a certain level, are eligible to receive sickness benefits. Payments are covered by the employer for the first 14 days, with periods exceeding 14 days covered by the Social Insurance Agency (and hence captured in national registers). Students can receive sickness absence benefits if they fulfil the eligibility requirements (having some income from work either before they started their studies, or during their studies). Students who have never received income from work are eligible for student aid, provided by The Swedish Board of Student Finance (CSN), if they become sick during their studies. However, as these payments are not sickness absence benefits per se they are not captured in the MiDAS database.	Individuals who have worked at least 74 hours for their employer in the 8 weeks prior to illness are eligible to receive sickness absence payments which are paid by their employer for the first 30 days, after which they are provided by the local authority (and hence captured in national registers). The local authority covers payments for the first 30 days for individuals who meet other requirements (e.g., having worked a minimum of 240 hours within the last 6 months a minimum of 40 hours for at least 5 of these months). Students who become unwell during their studies are not usually entitled to sickness absence benefits unless they are eligible via employment. Students are instead able to apply for a supplement to their study grant (SU-clip), but this is not captured in the SGDP register.
**Disability pension** [2]	Individuals aged 30-64 years can be granted permanent disability pension in Sweden, whilst those aged 19-29 years can receive time-restricted disability pension ('Aktivitetsersättning') if work capacity is reduced, or compulsory education is not completed. For both types, individuals can receive full-time or partial (part-time) disability pension.	Individuals aged ≥40 years are currently eligible to receive permanent disability pension in Denmark (førtidspension), those aged <40 years are also able to claim permanent disability pension in exceptional circumstances (where it must be deemed to be entirely evident that an individual cannot work). Whilst there are alternative, temporary payments available for those not able to work due to illness ('ressourceforløb') these are not considered disability pension per se as they aim to bring persons into the labour market gradually while offering financial support. Moreover, disability pension does not include partial disability pension ('delpension'), a financial compensation for those who wish to reduce their working hours without leaving the labour market altogether.

Note. The information above pertains to the current regulations in Sweden and Denmark which have undergone several changes in the past during the study period (2006-2018). Importantly, the current age restriction on receipt of permanent disability pension in Denmark (i.e., only granted to those aged < 40 years in exceptional circumstances) was introduced in 2013,[2] prior to this period, disability pension was available for individuals aged 18-65 years.

[1] The regulations pertain to individuals who receive salary from an employer, different regulations exist for those who are self-employed.

[2] Whilst it is possible to account for partial disability pension in Sweden by using net days (gross days * extent of DP), gross days were examined to enable alignment with Danish payment system (which, as noted above, does not include partial disability pension payments).

Table S3 Region of birth and duration of residence for non-refugee migrant and refugee populations

	Sweden				Denmark			
	**Non-refugee migrant (N = 772)**		**Refugee (N = 912)**		**Non-refugee migrant (N = 860)**		**Refugee (N = 509)**	
**Region of birth**								
Africa	119	(15.4)	216	(23.7)	113	(13.2)	107	(21.1)
Asia	192	(24.9)	128	(14.0)	192	(22.5)	116	(22.9)
West Asia	86	(11.1)	216	(23.7)	132	(15.5)	193	(38.1)
Europe	271	(35.1)	317	(34.8)	351	(41.1)	>80	(NR)
Other	104	(13.5)	35	(3.8)	66	(7.7)	<10	(NR)
**Duration of residence**								
0-5 years	128	(17.3)	104	(11.4)	50	(6.2)	19	(3.7)
6-10 years	204	(27.6)	168	(18.4)	126	(15.7)	120	(23.6)
11+ years	406	(55.0)	640	(70.2)	626	(78.1)	370	(72.7)

All data are shown as n (%). NR, not reported to prevent determination of cells with counts < 10.

Missing data: Region of birth, Danish non-refugee migrants (n = 6); Danish refugees (n = 2). Duration of residence, Swedish non-refugee migrants (n = 34); Danish non-refugee migrants (n = 58).

Table S4 Descriptive statistics for unemployment and work disability outcomes during the five-year follow-up among individuals with non-affective psychotic disorders in Sweden and Denmark

	**Sweden**								**Denmark**							
	**Total (n = 6750)**		**Swedish-born (n = 5066)**		**Non-refugee migrants (n = 772)**		**Refugees (n = 912)**		**Total (n = 8320)**		**Danish-born (n = 6951)**		**Non-refugee migrants (n = 860)**		**Refugees (n = 509)**	
**Unemployment days**																
Any days, N (%)	4387	(65.0)	3161	(62.4)	538	(69.7)	688	(75.4)	2586	(31.1)	2077	(29.9)	399	(39.4)	170	(33.4)
Number of days, median (IQR)	68	(0, 240)	50	(0, 208)	107	(0, 303)	135	(2, 362)	0	(0, 15)	0	(0, 12)	0	(0, 63)	0	(0, 30)
**Sickness absence days**																
Any days, N (%)	2618	(38.8)	2068	(40.8)	288	(37.3)	262	(28.7)	2155	(25.4)	1758	(25.3)	247	(28.7)	110	(21.6)
Number of days, median (IQR)	0	(0, 264)	0	(0, 293)	0	(0, 249)	0	(0, 101)	0	(0, 36)	0	(0, 34)	0	(0, 116)	0	(0, 0)
**Disability pension**																
Received, N (%)	2110	(31.3)	1588	(31.3)	215	(27.8)	307	(33.7)	1668	(20.0)	1345	(19.3)	174	(20.2)	149	(29.3)

Table S5 Sensitivity analyses for sickness absence with labour market income at baseline included as an additional covariate

	Sweden				Denmark			
	Zero-inflated component [a]		Count component [b]		Zero-inflated component [a]		Count component [b]	
	OR	(95% CI)	IRR	(95%CI)	OR	(95% CI)	IRR	(95%CI)
**Sickness absence**								
**Native-born**								
**Non-refugee migrant**	1.33	(1.11–1.61)	0.99	(0.88–1.11)	1.15	(0.96–1.39)	1.00	(0.90–1.10)
**Refugee**	1.36	(1.14–1.63)	1.07	(0.95–1.21)	1.24	(0.97–1.59)	0.95	(0.83–1.09)

OR: odds ratio; CI: confidence interval; IRR: incidence rate ratio. Adjusted models included age, gender, family situation, region of residence, education level, psychotic disorder diagnosis at first contact, previous treatment for any mental disorder, previous treatment for somatic conditions, calendar year at cohort entry, and labour market income at baseline.

[a] Zero-inflated component modelled using binary logistic regression to estimate the odds of excess/structural zero values.
[b] Count component modelled with negative binomial regression to estimate the rate of remaining (non-structural zero) days.

Table S6 Sensitivity analyses for unemployment and sickness absence outcomes accounting for receipt of disability pension during the five-year follow-up.

	Sweden								Denmark							
	Zero-inflated component [a]				Count component [b]				Zero-inflated component [a]				Count component [b]			
	Crude		Adjusted		Crude		Adjusted		Crude		Adjusted		Crude		Adjusted	
	OR	(95% CI)	OR	(95% CI)	IRR	(95%CI)	IRR	(95%CI)	OR	(95% CI)	OR	(95% CI)	IRR	(95%CI)	IRR	(95%CI)
**Unemployment days**																
**Native-born**																
**Non-refugee migrant**	0.83	(0.71–0.98)	0.76	(0.64–0.91)	1.28	(1.16–1.42)	1.23	(1.11–1.37)	0.67	(0.57–0.77)	0.78	(0.66–0.91)	1.29	(1.12–1.48)	1.20	(1.04–1.37)
**Refugee**	0.51	(0.43–0.61)	0.50	(0.42–0.60)	1.32	(1.21–1.44)	1.26	(1.15–1.38)	0.96	(0.79–1.17)	1.15	(0.93–1.42)	1.35	(1.11–1.64)	1.24	(1.03–1.50)
**Sickness absence days**																
**Native-born**																
**Non-refugee migrant**	1.17	(1.00–1.38)	1.49	(1.24–1.79)	0.99	(0.88–1.13)	0.99	(0.87–1.12)	0.83	(0.71–0.97)	1.15	(0.96–1.37)	0.98	(0.89–1.08)	0.97	(0.88–1.07)
**Refugee**	1.84	(1.57–2.16)	1.62	(1.35–1.93)	1.12	(0.98–1.28)	1.10	(0.96–1.26)	1.21	(0.98–1.51)	1.46	(1.15–1.85)	0.97	(0.84–1.12)	0.96	(0.83–1.10)

OR: odds ratio; CI: confidence interval; IRR: incidence rate ratio. Adjusted models included age, gender, family situation, region of residence, education level, psychotic disorder diagnosis at first contact, previous treatment for any mental disorder, previous treatment for somatic conditions, and calendar year at cohort entry.

[a] Zero-inflated component modelled using binary logistic regression to estimate the odds of excess/structural zero values. [b] Count component modelled with negative binomial regression to estimate the rate of remaining (non-structural zero) days.

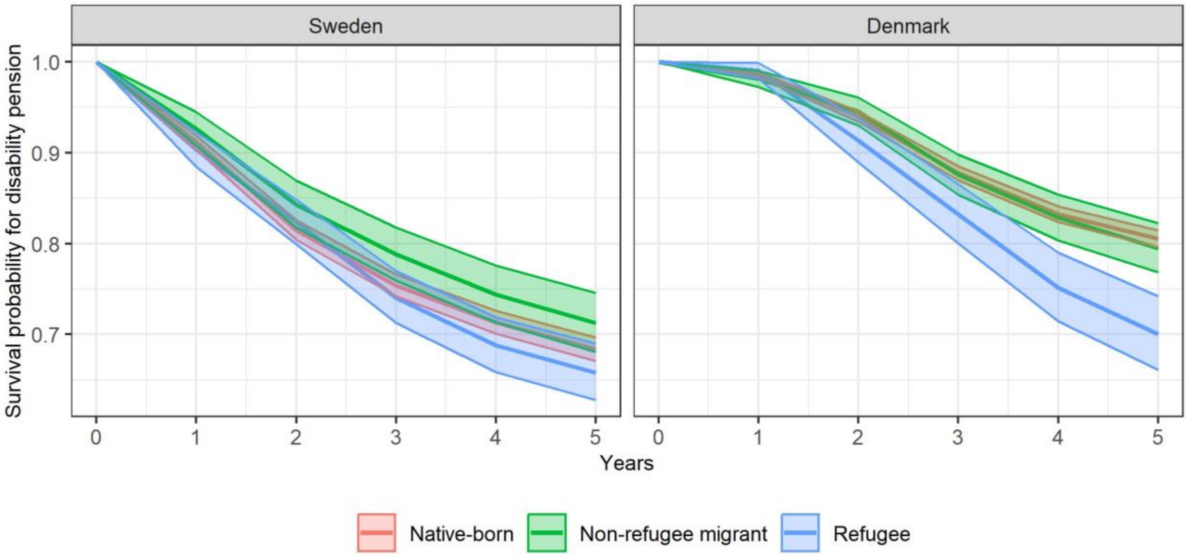

Figure S1. Kaplan-Meier estimated survival curves. Proportion not receiving disability pension during the five-year follow-up with 95% confidence intervals, by population group and country.

References

1 United Nations Statistics Division. Standard Country or Area Codes for Statistical Use (M49). New York.

2 Amby F, Schaldemose S, Christensen AB. Førtidspension fra kompensationstanke til udviklingsfokus. Tidsskrift for Arbejdsliv 2019;21:29-47.
